# Mortality of Philadelphia for July, August and September, 1852, Arranged from the Record Kept at the Health Office

**Published:** 1852-11

**Authors:** Wilson Jewell


*Mortality of Philadelphia for July, August, and September,* 1852, *arranged from the Record kept at the Health Office.* By Wilson Jewell, M. D.

In our last quarterly paper, published in the August number of the Examiner, we presented a few general remarks upon the Registration law of this State, and intimated, that the Board of Health had not only discontinued to publish their weekly statement of deaths, but had also declined receiving the certificates of births and deaths from physicians, under an impression that all matters appertaining thereto, had been, by the operation of the Registration law, transferred to the Register of Wills for the city and county of Philadelphia. As one of the results of this view of things connected with the law, the profession and the community at large were deprived of the benefit of a weekly statement of deaths during the month of July. This omission created much dissatisfaction and no little uneasiness in the public mind. The attention of the Board of Health being directed to the subject, they wisely gave it a careful revision, and found that the registration law neither infringed upon, nor interfered with, any part of the old law, under which they had been acting, "for establishing a health office, and to secure the city and port of Philadelphia from the introduction of pestilential and contagious diseases," passed January 29th, 1818, a part of the 26th section of which, reads as follows:—

"Whenever any person shall die in the city, districts or townships aforesaid, the physician or surgeon who shall have attended such person, as a physician or surgeon, during his or her last sickness, shall leave a note in writing signed with his name, with some one of the family in the house where such person shall have died, specifying the name and apparent age of the deceased, and the disease of which he or she shall have died.

"And that no sexton of any church, or other person having charge of any cemetery, vault or burying ground in the city and county aforesaid, shall permit any dead body to be interred therein, until he has received such note in writing so signed as aforesaid.

"The contents of which note in writing shall be entered by such sexton on a blank schedule to be furnished by the Clerk of the Health office, or such other person as the Board of Health shall direct, and delivered, together with the said schedule, on the Saturday of every week, to the Health office for publication, in such form as may be designated by the Board of Health."

It will be seen at once that the requirements and objects of the health law, are not only well defined, but entirely distinct and separate from the operations of the registration act. The one, affecting a single locality—its object, the preservation of the health of the city and the prevention of nuisances; while the other, looks to widely different and more general purposes, constituting an essential element towards a correct knowledge of the sanitary condition of the State at large, and is intimately allied to the physical and moral prosperity of its citizens.

The Board of Health, after a careful review of the whole subject, reconsidered their former action in the case, and resumed the duty enjoined upon them by the law of 1818, already referred to, of collecting and publishing a weekly statement of deaths occurring in the city and adjoining districts. By this decision we are enabled to continue our quarterly reports.

The returns of deaths made to the Board of Health for the third quarter of the present year, embracing a period extending from July 4th to October 2d, inclusive, making in all thirteen weeks or ninety-one days, amount in the aggregate to 2751.

This gives an increase of mortality of $4\frac{1}{2}$ per cent. over the second quarter of the year.

By deducting the still-born, the deaths from external causes, and from old age, the result shows the number of deaths from disease alone to be 2,497. Of these, the zymotic or epidemic class of diseases, amounting to 980, constitute more than one-third.

Dysentery has prevailed during the quarter to a very great extent, causing the death of 394 individuals, a higher per centage in proportion to the population, than has ever been recorded during any similar period in Philadelphia; constituting about sixteen per cent. of the aggregate of deaths from disease during the three months. For the three quarters of the year ending October 2d, the deaths from dysentery have amounted to 503.

Small pox is rapidly disappearing from our midst. Since June, only twenty-one deaths have been recorded, showing a falling off of eighty-five per cent. from the previous quarter.

The deaths from cholera infantum during July, August, and September, amounted to 273. It is gratifying to know, that the mortality from this fatal disease of infants is on the decline in this city since 1849. From Dr. Ruschenberger's annual report on epidemics, made to and published in the transactions of the College of Physicians, we find that the deaths in

1850 from cholera infantum for the 3d quarter were 436

1851 " " " " " " 344

And according to our present report for

1852, they only amount to 273

Presenting a reduction of deaths of about 40 per cent. in the two years.

Consumption of the lungs furnish 251 deaths for the quarter, being an increase of 37 over the third quarter of 1850, and nine over that of 1851, according to Dr. Ruschenberger's tables.

The most fatal diseases for the quarter have been as follows: dysentery, 394; cholera infantum, 273; consumption of the lungs, 251; convulsions, 139; marasmus, 131; debility, 114; amounting in the aggregate to 1302 deaths; 767, or more than one-half were among children under five years of age.

It appears that 1378, or more than 50 per cent. of the deaths recorded within the quarter, were among children under five, and nearly one-third of these took place within the first year of life.

### TABLE No. I.

*Deaths for the Third Quarter of* 1852, *classified.*

| | July. | August. | September. | MALE. J. | MALE. A. | MALE. S. | FEMALE. J. | FEMALE. A. | FEMALE. S. | Total. |
|---|---|---|---|---|---|---|---|---|---|---|
| 1. *Endemic and Contagious Diseases.* Zymotic or Epidemic . . | 333 | 364 | 283 | 161 | 175 | 140 | 172 | 189 | 143 | 980 |
| 2. *Uncertain or general seat.* Sporadic diseases . . . . . | 112 | 128 | 138 | 60 | 72 | 77 | 52 | 56 | 61 | 378 |
| 3. The Nervous System . . . | 170 | 126 | 139 | 98 | 67 | 71 | 72 | 59 | 68 | 435 |
| 4. Organs of Respiration . . . | 108 | 120 | 146 | 53 | 62 | 82 | 55 | 58 | 64 | 374 |
| 5. " Circulation . . . | 12 | 7 | 18 | 6 | 3 | 14 | 6 | 4 | 4 | 37 |
| 6. The Digestive Organs . . . | 55 | 61 | 70 | 30 | 40 | 32 | 25 | 21 | 38 | 186 |
| 7. The Urinary Organs . . . | 2 | 1 | | 2 | 1 | | | | | 3 |
| 8. The Organs of Generation . . | 9 | 12 | 9 | 2 | 3 | 2 | 7 | 9 | 7 | 30 |
| 9. " Locomotion . . | 4 | 4 | 2 | 3 | 3 | | 1 | 1 | 2 | 10 |
| 10. The Integumentary System . . | 2 | 2 | | 1 | | | 1 | 2 | | 4 |
| 11. Old Age . . . . . . | 9 | 8 | 13 | 2 | 2 | 3 | 7 | 6 | 10 | 30 |
| 12. External Causes . . . . . | 33 | 34 | 39 | 28 | 27 | 29 | 5 | 7 | 10 | 106 |
| Still Born . . . . . . | 36 | 38 | 44 | 17 | 21 | 25 | 19 | 17 | 19 | 118 |
| Unknown . . . . . . | 19 | 18 | 23 | 8 | 11 | 13 | 11 | 7 | 10 | 60 |
| | 904 | 923 | 924 | 471 | 487 | 488 | 433 | 436 | 436 | 2751 |

### TABLE No. II.

1. *Endemic and Contagious Diseases.—Zymotic or Epidemic.*

| | Male. | Female. | Under 1 yr. | 1 to 2. | 2 to 5. | 5 to 10. | 10 to 15. | 15 to 20. | 20 to 30. | 30 to 40. | 40 to 50. | 50 to 60. | 60 to 70. | 70 to 80. | 80 to 90. | 90 to 100. | 100 to 110. | Total. |
|---|---|---|---|---|---|---|---|---|---|---|---|---|---|---|---|---|---|---|
| Cholera Infantum . | 144 | 129 | 165 | 89 | 18 | 1 | | | | | | | | | | | | 273 |
| " Morbus . | 5 | 10 | 1 | 1 | | 1 | 1 | 2 | | 3 | 1 | 1 | 3 | 1 | | | | 15 |
| Croup . . . | 11 | 17 | 4 | 11 | 10 | 3 | | | | | | | | | | | | 28 |
| Diarrhœa . . . | 38 | 39 | 41 | 16 | 3 | | | | 2 | 4 | 1 | 3 | 3 | 2 | 2 | | | 77 |
| Dysentery . . | 182 | 212 | 58 | 64 | 53 | 34 | 9 | 12 | 34 | 38 | 29 | 24 | 18 | 17 | 2 | 1 | 1 | 394 |
| Erysipelas . . | 7 | 7 | 3 | | | | | | 3 | 2 | 3 | 2 | | | 1 | | | 14 |
| Fever . . . | 4 | 1 | 1 | 1 | | | | | 1 | 1 | 1 | | | | | | | 5 |
| " Bilious . . | 3 | 1 | | | | | | | | 2 | | 1 | 1 | | | | | 4 |
| " Catarrhal . | 1 | | | | 1 | | | | | | | | | | | | | 1 |
| " Congestive . | 3 | 1 | 1 | | | 1 | | 1 | 1 | | | | | | | | | 4 |
| " Hectic . . | 1 | 5 | 2 | | | 1 | | | 2 | 1 | | | | | | | | 6 |
| " Intermittent . | 1 | 1 | 1 | | | | | | | | | | 1 | | | | | 2 |
| " Remittent . | 9 | 6 | | 1 | 3 | | | 1 | 1 | 4 | 2 | 1 | 1 | | 1 | | | 15 |
| " Scarlet . . | 17 | 26 | 4 | 11 | 16 | 7 | 4 | | 1 | | | | | | | | | 43 |
| " Synocha . . | 1 | | | | | | | | | 1 | | | | | | | | 1 |
| " Typhus . . | 16 | 14 | | 2 | 1 | 1 | 1 | 3 | 10 | 7 | 5 | | | | | | | 30 |
| " Typhoid . . | 18 | 14 | | 1 | 1 | 1 | 1 | 2 | 5 | 7 | 1 | 3 | 7 | 2 | 1 | | | 32 |
| Hooping Cough . . | 3 | 1 | 3 | 1 | | | | | | | | | | | | | | 4 |
| Measles . . . | 4 | 2 | 1 | 3 | 2 | | | | | | | | | | | | | 6 |
| Small Pox. . . | 7 | 14 | 7 | 6 | 6 | 1 | | | 1 | | | | | | | | | 21 |
| Syphilis . . . | 1 | 1 | | | | | | | 1 | 1 | | | | | | | | 2 |
| Varioloid . . | | 3 | 1 | | 2 | | | | | | | | | | | | | 3 |
| | 476 | 504 | 293 | 207 | 116 | 51 | 16 | 21 | 62 | 71 | 43 | 35 | 34 | 22 | 7 | 1 | 1 | 980 |

2. *Uncertain or General Seat.—Sporadic Diseases.*

| | Male. | Female. | Under 1 yr. | 1 to 2. | 2 to 5. | 5 to 10. | 10 to 15. | 15 to 20. | 20 to 30. | 30 to 40. | 40 to 50. | 50 to 60. | 60 to 70. | 70 to 80. | 80 to 90. | 90 to 100. | 100 to 110. | Total. |
|---|---|---|---|---|---|---|---|---|---|---|---|---|---|---|---|---|---|---|
| Abscess . . . | | 1 | 1 | | | | | | | | | | | | | | | 1 |
| Cancer . . . | 1 | 3 | | | | | | | | 2 | 1 | | 1 | | | | | 4 |
| " Breast . . | | 3 | | | | | | | 1 | | 1 | | 1 | | | | | 3 |
| " Mouth . . | 1 | 1 | | | | 1 | | | | | 1 | | | | | | | 2 |
| Congestion . . . | 1 | | | | | | | | | 1 | | | | | | | | 1 |
| Cyanosis . . . | 4 | 1 | 3 | | | 1 | | | 1 | | | | | | | | | 5 |
| Debility . . . | 65 | 49 | 42 | 9 | 6 | 5 | 1 | 1 | 5 | 4 | 8 | 4 | 13 | 12 | 3 | 1 | | 114 |
| Disease of Mouth . | | 2 | 1 | 1 | | | | | | | | | | | | | | 2 |
| " of Neck . . | 1 | | | | | | | | | 1 | | | | | | | | 1 |
| Dropsy . . . | 18 | 17 | 3 | 3 | 3 | 1 | | | 2 | 4 | 4 | 4 | 8 | 3 | | | | 35 |
| Gangrene . . . | | 4 | | | | | | | | | | | 2 | 2 | | | | 4 |
| " of Foot . . | | 1 | | | | | | | | | | 1 | | | | | | 1 |
| " of Mouth . | | 1 | | 1 | | | | | | | | | | | | | | 1 |
| Hemorrhage . . | 3 | 1 | | 1 | | | | | 2 | | 1 | | | | | | | 4 |
| " from Umbilicus | | 1 | | | | | | | | 1 | | | | | | | | 1 |
| Inanition . . . | 15 | 15 | 28 | 1 | | | | | | | | | | | 1 | | | 30 |
| Inflammation . . | 2 | | 1 | 1 | | | | | | | | | | | | | | 2 |
| Marasmus . . . | 79 | 52 | 84 | 28 | 9 | 2 | 1 | 1 | 1 | | 2 | 1 | 1 | 1 | | | | 131 |
| Malformation . . | 5 | 2 | 7 | | | | | | | | | | | | | | | 7 |
| Melœna . . . | 1 | 1 | | | | | | | | | | 1 | 1 | | | | | 2 |
| Mortification . . | 1 | | | | 1 | | | | | | | | | | | | | 1 |
| Scrofula . . . | 5 | 3 | 2 | 1 | 2 | 1 | | | 2 | | | | | | | | | 8 |
| Tabes Mesenterica . | 6 | 8 | 5 | 4 | 3 | | | 2 | | | | | | | | | | 14 |
| Tumour . . . | | 2 | | | | | | 1 | | | | 1 | | | | | | 2 |
| " of Mouth . | 1 | | | | | | | | | | | 1 | | | | | | 1 |
| Ulceration · . . | | 1 | | | | | | | 1 | | | | | | | | | 1 |
| | 209 | 169 | 177 | 50 | 24 | 11 | 2 | 5 | 15 | 13 | 18 | 13 | 27 | 18 | 4 | 1 | | 378 |

3. *Of the Nervous System.*

| | Male. | Female. | Under 1 yr. | 1 to 2. | 2 to 5. | 5 to 10. | 10 to 15 | 15 to 20. | 20 to 30. | 30 to 40. | 40 to 50. | 50 to 60. | 60 to 70. | 70 to 80. | 80 to 90. | 90 to 100. | 100 to 110. | Total. |
|---|---|---|---|---|---|---|---|---|---|---|---|---|---|---|---|---|---|---|
| Abscess of Brain . . | | 1 | | | | | 1 | | | | | | | | | | | 1 |
| Apoplexy . . . | 9 | 11 | 1 | | | | 1 | | 2 | 1 | 1 | 3 | 3 | 4 | 4 | | | 20 |
| Catalepsy . . . | | 1 | | | | | | | 1 | | | | | | | | | 1 |
| Concussion of Brain . | 1 | | | | | | 1 | | | | | | | | | | | 1 |
| Congestion " . . | 21 | 10 | 10 | 5 | 3 | 2 | | 2 | 4 | | 1 | | 3 | 1 | | | | 31 |
| Convulsions . . | 62 | 77 | 79 | 29 | 14 | 5 | 1 | | 6 | 1 | 1 | 1 | 2 | | | | | 139 |
| " Puerperal . | | 5 | | | | | | 1 | 1 | 3 | | | | | | | | 5 |
| Coup de Soleil . . | 2 | | | | | | | | 1 | 1 | | | | | | | | 2 |
| Cramp . . . | 1 | | 1 | | | | | | | | | | | | | | | 1 |
| Disease of Brain . . | 14 | 8 | 9 | 7 | 3 | | | | | | 1 | 2 | | | | | | 22 |
| Dropsy " . . | 41 | 27 | 27 | 22 | 9 | 5 | | 1 | 1 | | 2 | | 1 | | | | | 68 |
| Effusion " . . | 16 | 8 | 14 | 4 | 1 | 1 | | | 2 | | | 1 | | 1 | | | | 24 |
| Enlargement " . . | | 1 | 1 | | | | | | | | | | | | | | | 1 |
| Fever " . . | 1 | | 1 | | | | | | | | | | | | | | | 1 |
| Inflammation " . . | 41 | 31 | 20 | 18 | 13 | 6 | 3 | 5 | | 2 | 3 | 1 | 1 | | | | | 72 |
| Epilepsy . . . | 3 | 1 | | | 1 | | | | 1 | | 1 | | 1 | | | | | 4 |
| Mania . . . | 3 | 4 | | | | | | | 1 | 1 | 3 | 1 | 1 | | | | | 7 |
| " a potu . . | 6 | 1 | | | | | | | | 3 | | 2 | 1 | 1 | | | | 7 |
| Palsy . . . . | 9 | 12 | | | 1 | 1 | | | 2 | | 1 | 1 | 6 | 4 | 5 | | | 21 |
| Softening of Brain . | 3 | 1 | | | | | | | 1 | | 2 | 1 | | | | | | 4 |
| Tetanus . . . | 3 | | 1 | | | | | | 1 | | | 1 | | | | | | 3 |
| | 236 | 199 | 164 | 85 | 45 | 20 | 7 | 9 | 24 | 12 | 16 | 14 | 19 | 11 | 9 | | | 435 |

### 4. *Organs of Respiration.*

| | Male. | Female. | Under 1 yr. | 1 to 2. | 2 to 5. | 5 to 10. | 10 to 15. | 15 to 20. | 20 to 30. | 30 to 40. | 40 to 50. | 50 to 60. | 60 to 70. | 70 to 80. | 80 to 90. | 90 to 100. | 100 to 110. | Total. |
|---|---|---|---|---|---|---|---|---|---|---|---|---|---|---|---|---|---|---|
| Abscess of Lungs . | 1 | 1 | | 1 | | | | | | | | | 1 | | | | | 2 |
| Asthma . . . | 2 | 2 | 1 | | | | | | | 1 | 1 | | | 1 | | | | 4 |
| Cancer of Larynx . | | 1 | | | | | | | | 1 | | | | | | | | 1 |
| Congestion of Lungs . | 5 | 8 | 4 | 1 | 2 | | | | 1 | 4 | 1 | | | | | | | 13 |
| Consumption " . | 124 | 127 | 7 | 5 | 8 | 7 | 5 | 14 | 84 | 53 | 36 | 17 | 12 | 3 | | | | 251 |
| Disease of Lungs . | 2 | 1 | | | | | | | 1 | 3 | | | | | | | | 3 |
| " Chest . . | 1 | | | | | | | | | | 1 | | | | | | | 1 |
| Dropsy of Chest . . | 8 | 2 | 1 | | 3 | 1 | | | 1 | 1 | | 1 | 1 | 1 | | | | 10 |
| Effusion " . . | | 1 | | | | | | | | | | | 1 | | | | | 1 |
| " Lungs . . | 1 | 1 | | | | 1 | | | | 1 | | | | | | | | 2 |
| Hemorrhage " . . | 3 | | | | | | | | | 2 | 1 | | | | | | | 3 |
| Inflammation Bronchiæ | 14 | 7 | 7 | 5 | | 1 | | | 3 | 3 | 1 | 1 | | | | | | 21 |
| " Chest . | 1 | | 1 | | | | | | | | | | | | | | | 1 |
| " Larynx . | 1 | | | | | | 1 | | | | | | | | | | | 1 |
| " Lungs . | 22 | 19 | 16 | 5 | 6 | 2 | 2 | | 6 | 2 | | 1 | | | 1 | | | 41 |
| " Pleura . | 1 | 2 | | | | | | | | | | 3 | | | | | | 3 |
| " Throat . | 2 | 1 | 1 | | | | | 1 | | | | | | 1 | | | | 3 |
| Obstruction of Trachea | | 1 | | 1 | | | | | | | | | | | | | | 1 |
| Tuberculosis . . | 9 | 3 | | 4 | | 1 | | | 2 | 1 | 2 | 1 | 1 | | | | | 12 |
| | 197 | 177 | 38 | 22 | 19 | 13 | 8 | 15 | 98 | 71 | 43 | 24 | 16 | 6 | 1 | | | 374 |

### 5. *Organs of Circulation.*

| | Male. | Female. | Under 1 yr. | 1 to 2. | 2 to 5. | 5 to 10. | 10 to 15. | 15 to 20. | 20 to 30. | 30 to 40. | 40 to 50. | 50 to 60. | 60 to 70. | 70 to 80. | 80 to 90. | 90 to 100. | 100 to 110. | Total. |
|---|---|---|---|---|---|---|---|---|---|---|---|---|---|---|---|---|---|---|
| Angina Pectoris . | 1 | | | | | | | | 1 | | | | | | | | | 1 |
| Disease of Heart . . | 12 | 8 | 2 | 1 | 2 | | | | 3 | 5 | 4 | 2 | 1 | | | | | 20 |
| Dropsy " . . | 4 | 3 | 1 | | 1 | | 1 | | | 1 | | 1 | 1 | 1 | | | | 7 |
| Enlargement of Heart . | 4 | 2 | | | | | 1 | | 1 | | 1 | 2 | 1 | | | | | 6 |
| Inflammation " . | 1 | | | | | | | | | | | 1 | | | | | | 1 |
| Ossification " . | 1 | 1 | | | | | | | | | | | | | 2 | | | 2 |
| | 23 | 14 | 3 | 1 | 3 | | 2 | | 5 | 6 | 5 | 6 | 3 | 1 | 2 | | | 37 |

### 6. *Digestive Organs.*

| | Male. | Female. | Under 1 yr. | 1 to 2. | 2 to 5. | 5 to 10. | 10 to 15. | 15 to 20. | 20 to 30. | 30 to 40. | 40 to 50. | 50 to 60. | 60 to 70. | 70 to 80. | 80 to 90. | 90 to 100. | 100 to 110. | Total. |
|---|---|---|---|---|---|---|---|---|---|---|---|---|---|---|---|---|---|---|
| Abscess of Liver . . | 2 | | | | | | | | 1 | 1 | | | | | | | | 2 |
| Abdominal Dropsy . | 11 | 3 | | | 1 | | | | 1 | 6 | 2 | 1 | 2 | 1 | | | | 14 |
| Cancer, Liver . . | | 1 | | | | | | | | | | 1 | | | | | | 1 |
| " Stomach . . | | 1 | | | | | | | | | | | 1 | | | | | 1 |
| Cancrum Oris . . | 1 | | | 1 | | | | | | | | | | | | | | 1 |
| Colic . . . . . | 1 | 1 | 2 | | | | | | | | | | | | | | | 2 |
| Cirrhosis of Liver . | 4 | 1 | | | | | | | 1 | 1 | 1 | | | 2 | | | | 5 |
| Congestion of Bowels . | 2 | | 1 | | | | | | | | | 1 | | | | | | 2 |
| Constipation . . | 1 | | | | | | | | 1 | | | | | | | | | 1 |
| Consumption of Bowels | | 3 | | | 2 | | | | | 1 | | | | | | | | 3 |
| Disease of Abdomen . | 1 | | 1 | | | | | | | | | | | | | | | 1 |
| " Bowels . | 1 | 1 | 2 | | | | | | | | | | | | | | | 2 |
| " Liver . . | 2 | 2 | 1 | | | | | | | 1 | 2 | | | | | | | 4 |
| " Stomach . | 2 | 2 | 2 | | | | | | 1 | | | 1 | | | | | | 4 |
| " Stomach& bowels | 1 | | 1 | | | | | | | | | | | | | | | 1 |
| Hemorrhage of Bowels | 1 | 1 | 1 | | | | | | | 1 | | | | | | | | 2 |
| Hernia, strangulated . | 1 | | | | | | | | | | | 1 | | | | | | 1 |
| Induration of Liver . | | 1 | | | | | | | | | | | | 1 | | | | 1 |
| Inflammation of Bowels | 1 | 2 | | 1 | | | | | | 2 | | | | | | | | 3 |
| " Liver . | 4 | 5 | 1 | 1 | | 1 | | | 2 | | 1 | 1 | 2 | | | | | 9 |
| " Peritoneum . | 2 | 7 | | | | 1 | 2 | 1 | 3 | | 1 | | | | 1 | | | 9 |
| Inflam. Stomach & Bowels | 45 | 39 | 15 | 14 | 12 | 3 | 1 | 3 | 7 | 7 | 7 | 7 | 5 | 2 | 1 | | | 84 |
| Intussusception . . | | 2 | | | | | | | 2 | | | | | | | | | 2 |
| Jaundice . . . . | 3 | 2 | 3 | | | | | | | | | | 2 | | | | | 5 |
| Mortification Bowels . | 2 | | | | 1 | | 1 | | | | | | | | | | | 2 |
| Obstruction Bowels . | 1 | 1 | | | | | | 1 | | | | | | | 1 | | | 2 |
| Rupture " . | 1 | | | | | 1 | | | | | | | | | | | | 1 |
| Scirrhus Mesentery . | 1 | | | | | | | | | | | | 1 | | | | | 1 |
| " Stomach . | | 1 | | | | | | | | | 1 | | | | | | | 1 |
| Softening Stomach . | | 1 | | | 1 | | | | | | | | | | | | | 1 |
| Teething . . . . | 2 | 4 | 2 | 4 | | | | | | | | | | | | | | 6 |
| Ulceration of Bowels . | 4 | | | | | | | | | 1 | 2 | | 1 | | | | | 4 |
| " Stomach . | 1 | 1 | | | | | | | | 1 | | | 1 | | | | | 2 |
| " Throat . | 3 | 2 | 1 | 1 | | 2 | | 1 | | | | | | | | | | 5 |
| Worms . . . . | 1 | | | | | 1 | | | | | | | | | | | | 1 |
| | 102 | 84 | 33 | 22 | 17 | 9 | 4 | 6 | 19 | 22 | 17 | 13 | 15 | 6 | 3 | | | 186 |

### 7. *The Urinary Organs.*

| | Male. | Female. | Under 1 yr. | 1 to 2. | 2 to 5. | 5 to 10. | 10 to 15. | 15 to 20. | 20 to 30. | 30 to 40. | 40 to 50. | 50 to 60. | 60 to 70. | 70 to 80. | 80 to 90. | 90 to 100. | 100 to 110. | Total. |
|---|---|---|---|---|---|---|---|---|---|---|---|---|---|---|---|---|---|---|
| Albuminuria . . | 1 | | | | | | | | | | 1 | | | | | | | 1 |
| Inflam. of Kidneys . | 2 | | | | | | | | | | | | 1 | 1 | | | | 2 |
| | 3 | | | | | | | | | | 1 | | 1 | 1 | | | | 3 |

### 8. *Organs of Generation.*

| | Male. | Female. | Under 1 yr. | 1 to 2. | 2 to 5. | 5 to 10. | 10 to 15. | 15 to 20. | 20 to 30. | 30 to 40. | 40 to 50. | 50 to 60. | 60 to 70. | 70 to 80. | 80 to 90. | 90 to 100. | 100 to 110. | Total. |
|---|---|---|---|---|---|---|---|---|---|---|---|---|---|---|---|---|---|---|
| Abortion . . . | | 1 | | | | | | | 1 | | | | | | | | | 1 |
| Cancer Uterus . . | | 2 | | | | | | | | | 1 | | 1 | | | | | 2 |
| Disease of Urinary Organs | 1 | | | | | | | | | | | | | 1 | | | | 1 |
| " Uterus . | | 2 | | | | | | | | | 1 | | 1 | | | | | 2 |
| Hysteritis . . . | | 1 | | | | | | | | | | | 1 | | | | | 1 |
| Hemorrhage from Uterus | | 4 | | | | | | | 1 | 2 | 1 | | | | | | | 4 |
| Inflammation " | | 6 | | | | | | | 4 | 2 | | | | | | | | 6 |
| Inflam. of Bladder . | 2 | | 1 | | | | | | | | | | | 1 | | | | 2 |
| Orchitis . . . | 1 | | | | | | | | | | 1 | | | | | | | 1 |
| Prostatic Disease . | 1 | | | | | | | | | | | 1 | | | | | | 1 |
| Puerperal Fever . . | | 5 | | | | | | | 2 | 2 | 1 | | | | | | | 5 |
| " Mania . . | | 1 | | | | | | | | 1 | | | | | | | | 1 |
| Strangury . . . | 1 | | | | | | | | | | | | | 1 | | | | 1 |
| Ulceration of Urethra . | 1 | | | | | | | | | | 1 | | | | | | | 1 |
| " Uterus . | | 1 | | | | | | | | | 1 | | | | | | | 1 |
| | 7 | 23 | 1 | | | | | | 8 | 7 | 7 | 1 | 3 | 3 | | | | 30 |

### 9. *Organs of Locomotion.*

| | Male. | Female. | Under 1 yr. | 1 to 2. | 2 to 5. | 5 to 10. | 10 to 15. | 15 to 20. | 20 to 30. | 30 to 40. | 40 to 50. | 50 to 60. | 60 to 70. | 70 to 80. | 80 to 90. | 90 to 100. | 100 to 110. | Total. |
|---|---|---|---|---|---|---|---|---|---|---|---|---|---|---|---|---|---|---|
| Caries of Spine . . | 1 | | | | | | | | | | 1 | | | | | | | 1 |
| Disease of Knee Joint . | 1 | | | | | | | | 1 | | | | | | | | | 1 |
| " Hip Joint . | | 1 | | | | | | 1 | | | | | | | | | | 1 |
| Inflam. of Spinal Marrow | 1 | 1 | 1 | | | | | | | 1 | | | | | | | | 2 |
| Rheumatism . . | 3 | 2 | | | | | | | 2 | 1 | | | 1 | 1 | | | | 5 |
| | 6 | 4 | 1 | | | | | 1 | 3 | 2 | 1 | | 1 | 1 | | | | 10 |

### 10. *The Integumentary System.*

| | Male. | Female. | Under 1 yr. | 1 to 2. | 2 to 5. | 5 to 10. | 10 to 15. | 15 to 20. | 20 to 30. | 30 to 40. | 40 to 50. | 50 to 60. | 60 to 70. | 70 to 80. | 80 to 90. | 90 to 100. | 100 to 110. | Total. |
|---|---|---|---|---|---|---|---|---|---|---|---|---|---|---|---|---|---|---|
| Carbuncle . . . | | 2 | 1 | | | | | | | | | | 1 | | | | | 2 |
| Purpura Hemorrhagica | | 1 | 1 | | | | | | | | | | | | | | | 1 |
| Scorbutus . . . | 1 | | | | | | | | | | 1 | | | | | | | 1 |
| | 1 | 3 | 2 | | | | | | | | 1 | | 1 | | | | | 1 |

### 11. *Old Age.*

| | | | | | | | | | | | | | | | | | | |
|---|---|---|---|---|---|---|---|---|---|---|---|---|---|---|---|---|---|---|
| Old Age . . . | 7 | 23 | | | | | | | | | | | 1 | 9 | 13 | 5 | 2 | 30 |

12. *From External Causes.*

| | Male. | Female | Under 1 yr. | 1 to 2. | 2 to 5. | 5 to 10. | 10 to 15. | 15 to 20. | 20 to 30. | 30 to 40. | 40 to 50. | 50 to 60. | 60 to 70. | 70 to 80. | 80 to 90. | 90 to 100. | 100 to 110. | Total. |
|---|---|---|---|---|---|---|---|---|---|---|---|---|---|---|---|---|---|---|
| Asphyxia . . . | 4 | 2 | 5 | | | | | | 1 | | | | | | | | | 6 |
| Burns and Scalds . . | 2 | 4 | 1 | 1 | 1 | 2 | | | | | 1 | | | | | | | 6 |
| Casualties . . . | 30 | 4 | 2 | 1 | 1 | 2 | 1 | 3 | 8 | 5 | 5 | 2 | 2 | | 1 | 1 | | 34 |
| Drowned . . . | 30 | 3 | | | 3 | 2 | 3 | 1 | 6 | 2 | 8 | 5 | | 3 | | | | 33 |
| Fracture Pelvis . . | 1 | | | | | | | | 1 | | | | | | | | | 1 |
| " Skull . . | 2 | | | | | | 1 | | 1 | | | | | | | | | 2 |
| Intemperance . . | 12 | 7 | | | | | | | 3 | 5 | 3 | 4 | 4 | | | | | 19 |
| Suicide . . . | 1 | 2 | | | | | | | 3 | | | | | | | | | 3 |
| Violence . . . | 1 | | 1 | | | | | | | | | | | | | | | 1 |
| Wound . . . | 1 | | | | | | | | 1 | | | | | | | | | 1 |
| | 84 | 22 | 9 | 2 | 5 | 6 | 5 | 4 | 24 | 12 | 17 | 11 | 6 | 3 | 1 | 1 | | 106 |
| Unknown . . . | 32 | 28 | 23 | 3 | 3 | | | 2 | 7 | 4 | 10 | 3 | 2 | 2 | | 1 | | 60 |
| Still Born . . . | 63 | 55 | 118 | | | | | | | | | | | | | | | 118 |

TABLE NO. 3.

*Deaths for the Third quarter, at fifteen distinct periods of life.*

| | |
|---|---|
| Under 1 year, . . . . . . | 744 |
| 1 to 2 . . . . . . . | 392 |
| 2 to 5 . . . . . . . | 232 |
| 5 to 10 . . . . . . . | 110 |
| 10 to 15 . . . . . . . | 44 |
| 15 to 20 . . . . . . . | 63 |
| 20 to 30 . . . . . . . | 265 |
| 30 to 40 . . . . . . . | 220 |
| 40 to 50 . . . . . . . | 179 |
| 50 to 60 . . . . . . . | 120 |
| 60 to 70 . . . . . . . | 129 |
| 70 to 80 . . . . . . . | 83 |
| 80 to 90 . . . . . . . | 40 |
| 90 to 100 . . . . . . . | 9 |
| 100 to 110 . . . . . . . | 3 |
| | 2633 |
| Still Born . . . . . . . | 118 |
| Total, | 2751 |

The deaths in the Blockley Almshouse for the quarter, amounted to 225; among people of color, 186; and from the country, 43, as follows:

| | Almshouse. | Blacks. | Country. |
|---|---|---|---|
| July, . . . . . | 69 | 63 | 18 |
| August, . . . . . | 71 | 51 | 17 |
| September, . . . . | 85 | 72 | 8 |
| Total, | 225 | 186 | 43 |

